# Is developmental plasticity triggered by DNA methylation changes in the invasive cane toad (*Rhinella marina*)?

**DOI:** 10.1002/ece3.11127

**Published:** 2024-03-06

**Authors:** Boris Yagound, Roshmi R. Sarma, Richard J. Edwards, Mark F. Richardson, Carlos M. Rodriguez Lopez, Michael R. Crossland, Gregory P. Brown, Jayna L. DeVore, Richard Shine, Lee A. Rollins

**Affiliations:** ^1^ Evolution & Ecology Research Centre, Biological, Earth and Environmental Sciences University of New South Wales Sydney New South Wales Australia; ^2^ Centre for Integrative Ecology, School of Life and Environmental Sciences Deakin University Geelong Victoria Australia; ^3^ Evolution & Ecology Research Centre, School of Biotechnology and Biomedical Sciences University of New South Wales Sydney New South Wales Australia; ^4^ Minderoo OceanOmics Centre at UWA, Oceans Institute Deakin University Geelong Victoria Australia; ^5^ Deakin Genomics Research and Discovery Facility Deakin University, Locked Bag Geelong VIC Australia; ^6^ School of Agriculture, Food and Wine, Waite Research Institute The University of Adelaide Glen Osmond South Australia Australia; ^7^ Environmental Epigenetics and Genetics Group, Department of Horticulture College of Agriculture, Food and Environment, University of Kentucky Lexington Kentucky USA; ^8^ School of Life and Environmental Sciences University of Sydney Sydney New South Wales Australia; ^9^ Department of Biological Sciences Macquarie University Sydney New South Wales Australia; ^10^ UMR 241 EIO University of French Polynesia, IFREMER, ILM, IRD Faa’a Tahiti French Polynesia

**Keywords:** *Bufo marinus*, cane toad, development, DNA methylation, epigenetics, phenotypic plasticity

## Abstract

Many organisms can adjust their development according to environmental conditions, including the presence of conspecifics. Although this developmental plasticity is common in amphibians, its underlying molecular mechanisms remain largely unknown. Exposure during development to either ‘cannibal cues’ from older conspecifics, or ‘alarm cues’ from injured conspecifics, causes reduced growth and survival in cane toad (*Rhinella marina*) tadpoles. Epigenetic modifications, such as changes in DNA methylation patterns, are a plausible mechanism underlying these developmental plastic responses. Here we tested this hypothesis, and asked whether cannibal cues and alarm cues trigger the same DNA methylation changes in developing cane toads. We found that exposure to both cannibal cues and alarm cues was associated with local changes in DNA methylation patterns. These DNA methylation changes affected genes putatively involved in developmental processes, but in different genomic regions for different conspecific‐derived cues. Genetic background explains most of the epigenetic variation among individuals. Overall, the molecular mechanisms triggered by exposure to cannibal cues seem to differ from those triggered by alarm cues. Studies linking epigenetic modifications to transcriptional activity are needed to clarify the proximate mechanisms that regulate developmental plasticity in cane toads.

## Graphical abstract

Exposure during development to either cannibal cues from older conspecifics, or alarm cues from injured conspecifics, causes reduced growth and survival in cane toads. Exposure to both cannibal cues and alarm cues induces local changes in DNA methylation patterns but in different genomic regions. Developmental plasticity in cane toads seems to be triggered by distinct molecular mechanisms for each conspecific cue.

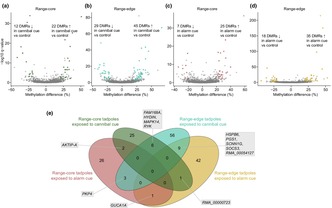

## 1 INTRODUCTION

Developmental plasticity is the ability of a single genotype to give rise to a range of phenotypes in different environments. Plasticity can be adaptive when environmental conditions are predictable and can involve both short‐ and long‐term changes in physiology, morphology, life‐history traits and behaviour (Sultan, 2003; West‐Eberhard, 2003). Although studies on the underlying molecular mechanisms of plasticity are burgeoning, these mechanisms remain poorly understood (Gilbert & Epel, 2015; Lafuente & Beldade, 2019; Sommer, 2020).

Amphibians are good models to study molecular mechanisms of phenotypic plasticity because the growth and development of their aquatic larvae are susceptible to environmental factors such as food availability, predator exposure, conspecific density, and pond drying (Denver, 2021; Newman, 1992; Wilbur & Collins, 1973). The invasive cane toad, *Rhinella marina*, is a good exemplar of a species capable of altering its developmental trajectory in response to environmental conditions (Lever, 2001). Female cane toads lay large clutches (up to several tens of thousands of eggs) asynchronously in ponds, resulting in overlapping cohorts of developing offspring where egg and larvae densities can reach extreme levels (Alford et al., 1995; DeVore, Crossland, & Shine, 2021). Cannibalism of eggs and hatchlings by older tadpoles is common in these breeding ponds, such that survival of newly‐laid eggs to the free‐swimming tadpole stage is often <1% (Alford et al., 1995; DeVore, Crossland, & Shine, 2021).

Interestingly, the presence of conspecifics affects cane toad larval development in two contexts (Crossland & Shine, 2012; Hagman et al., 2009). First, hatchlings are affected by ‘cannibal cues’ associated with the approach of older, cannibalistic tadpoles (Clarke et al., 2015; Crossland & Shine, 2011, 2012; DeVore, Crossland, & Shine, 2021). Exposure to cannibal cues causes hatchlings to accelerate development, with significant carry‐over effects during the subsequent tadpole stage: decreased tadpole survival, decreased body mass and body size (i.e., growth), reduced tooth row keratinization, increased swimming behaviour and repression of feeding behaviour (Clarke et al., 2015; Crossland & Shine, 2012; DeVore, Crossland, & Shine, 2021; DeVore, Crossland, Shine, & Ducatez, 2021; McCann et al., 2020). Second, injured tadpoles release an ‘alarm cue’, reflecting a predation risk, that elicits immediate avoidance by conspecifics (Hagman & Shine, 2008). Chronic exposure to alarm cues during tadpole development decreases tadpole survival, reduces growth at metamorphosis, increases the size of parotoid glands, and can reduce development rate (Crossland et al., 2019; Hagman et al., 2009; Hagman & Shine, 2009).

Both alarm cues and cannibal cues thus induce developmental plastic responses and reduce growth and survival in cane toad tadpoles. Moreover, both cues likely have long‐term detrimental effects post‐metamorphosis, affecting adult survival against cannibalism (Pizzatto & Shine, 2008), predation (Ward‐Fear et al., 2012), parasitism (Kelehear et al., 2009) and desiccation (Child et al., 2008). Finally, these cues appear mechanistically associated with one another, because exposure to alarm cues can have intergenerational effects by increasing the potency of cannibal cues in the next generation (Sarma et al., 2021).

In this study, we asked whether exposure to cannibal cues and exposure to alarm cues might trigger the same molecular mechanisms. Specifically, we tested (i) whether exposure to conspecific cues triggers changes in DNA methylation patterns in cane toad tadpoles that might then induce developmental plasticity, and (ii) whether both cannibal cues and alarm cues trigger the same epigenetic modifications. We focused on DNA methylation, because this epigenetic mechanism can influence transcriptional activity on the one hand (Jaenisch & Bird, 2003), and can be affected by environmental factors on the other (Dowen et al., 2012; Radford et al., 2014), and because studies of cane toads have shown that exposure to alarm cues changes DNA methylation patterns in tadpoles (Sarma et al., 2020, 2021). Changes in DNA methylation are thus a plausible molecular mechanism that might underlie developmental plasticity in cane toads.

## 2 MATERIALS AND METHODS

### 2.1 Toads

**FIGURE 1:**
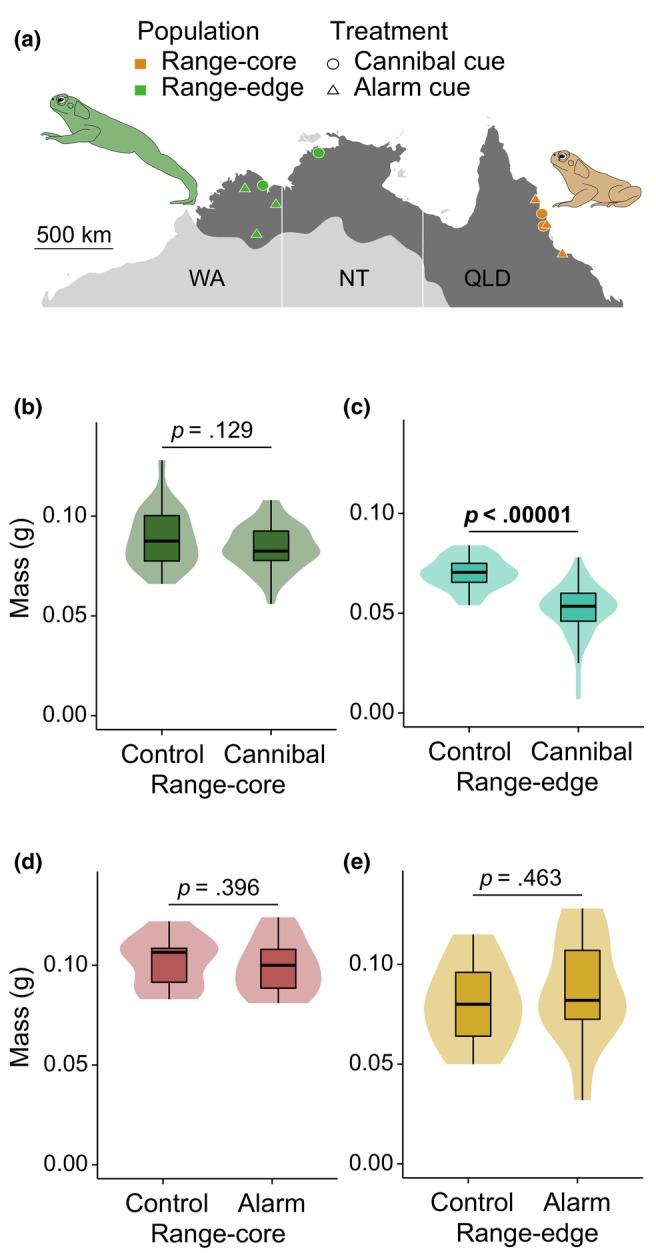
Populations used and effect of exposure to conspecific cues on tadpole growth. (a) Location of samples. Samples used in the cannibal cue experiment originated from localities depicted by circles. Samples used in the alarm cue experiment originated from localities depicted by triangles. Orange and green localities respectively correspond to range‐core and range‐edge populations. NT, Northern Territory; QLD, Queensland; WA, Western Australia. The shaded area represents the cane toad's Australian invasive range. (b, c) Mass (g) of tadpoles exposed to cannibal cues and controls from (b) range‐core and (c) range‐edge populations, and of tadpoles exposed to alarm cues and controls from (d) range‐core and (e) range‐edge populations. Violin plots represent median, interquartile range (IQR), 1.5 × IQR, and kernel density plot. Significant *p*‐values (LMMs) are highlighted in bold.

We collected adult cane toads from two genetically distinct populations (Selechnik et al., 2019) within the Australian invasive range: ‘range core’ and ‘range edge’. We collected range‐core toads from five localities in Queensland: Townsville, Mission Beach, Port Douglas, Innisfail and Tully. We collected range‐edge toads from one locality in the Northern Territory, Middle Point, and from four localities in Western Australia: Doongan, Lake Argyle, Mary Pool and Oombulgarri (Figure 1a). We transported all collected toads to our field station in Middle Point, Northern Territory, where they were maintained in outdoor enclosures with refugia, water and a constant food supply. We subcutaneously injected two pairs of toads from each locality with synthetic gonadotropin to induce spawning (see DeVore, Crossland, and Shine (2021) for details). We then kept eggs in aerated holding tanks for 72 h to ensure successful fertilisation.

### 2.2 Experimental design

In this study, we compared the effect of conspecific exposure on DNA methylation during development in two contexts: exposure to cannibal cues and exposure to alarm cues.

### 2.3 Cannibal cue experiment

In this experiment, we exposed focal hatchlings from eight clutches (i.e., 4 range‐core and 4 range‐edge clutches) to three treatments: (1) exposure to conspecific tadpoles (i.e., cannibal cues) from clutch *i*, (2) exposure to conspecific tadpoles from clutch *j*, (3) control (no conspecific tadpoles added). Clutches *i* and *j* were raised from clutches from the same locality as the focal hatchlings but were included so that we could measure the impact of genotype on the effect of cannibal cues. We used 1 L‐treatment tanks filled with 750 mL of water from a local aquifer. In the cannibal cue treatments, we first added three captive‐raised tadpoles [developmental stage 30–35 (Gosner, 1960)] to each tank, separated by a 1 × 1 mm fly screen mesh from the compartment where focal hatchlings were placed. This allowed chemical cannibal cues to diffuse throughout the container but prevented cannibalism (Crossland & Shine, 2012). Several hours after conspecific tadpoles were added, we randomly allocated five hatchlings [developmental stage 16/17, i.e., approximately 1 day post‐hatching (Gosner, 1960)] to each treatment tank. We replicated each treatment three times. We removed the conspecific tadpoles after 24 h and left the developing hatchlings for a further 24 h, by which time they had developed into free‐swimming, feeding tadpoles (stage 25). We then transferred the stage 25 tadpoles to new tanks and fed them blended Hikari algae wafers (Kyorin, Japan) ad libitum, with fresh water changes every 3 days. Ten days later, we humanely euthanised all tadpoles (stage ~32) and three tadpoles from each tank were blotted, weighed, and then frozen prior to DNA extraction.

### 2.4 Alarm cue experiment

This experiment was described in Sarma et al. (2020). Briefly, we randomly allocated two hatchlings from each clutch to two treatments: (1) exposure to conspecific alarm cues (*n* = 11 clutches, each region), (2) control (*n* = 10 clutches, range core; *n* = 9 clutches, range edge). We used hatchlings from the same clutches for each of these two treatments, unless mortality prevented us from doing so (i.e., three cases where only one hatchling was available and was allocated to the alarm cue treatment). In the alarm cue treatment, we added to each tank 4 mL of water containing the freshly‐macerated bodies of two conspecific tadpoles (Hagman et al., 2009) on each of 10 consecutive days (days 7–16 post spawning). In the control treatment, we added 4 mL of non‐chlorinated water on 10 consecutive days. Two days later, we humanely euthanised two 18‐days old tadpoles (stage ~35) per tank, weighed them, and preserved them in RNALater; one of these individuals was used for DNA methylation analysis.

### 2.5 Reduced‐representation bisulfite sequencing

We extracted DNA from whole tadpoles using a Gentra Puregene Kit (Qiagen) according to the manufacturer's instructions. We prepared reduced‐representation bisulfite sequencing (RRBS) libraries using 100 ng of genomic DNA per sample with the Ovation RRBS Methyl‐Seq Kit (NuGEN Technologies, San Carlos, USA). Libraries (100 bp single‐end) were sequenced on a NovaSeq 6000 S2 flowcell platform (Illumina, San Diego, USA). RRBS library preparation and sequencing were conducted at the Ramaciotti Centre for Genomics (UNSW, Sydney, Australia).

### 2.6 DNA methylation analyses

We used FastQC 0.11.8 (Andrews, 2010) to assess the quality of the reads. We trimmed adapter sequences and low‐quality reads using Trim Galore 6.5 (Krueger, 2015). We then mapped the remaining reads to the cane toad genome (Edwards et al., 2018) using Bismark 0.22.3 (Krueger & Andrews, 2011) with HISAT2 2.1.0 (Kim et al., 2019). We extracted methylation status for each CpG using Bismark. We carried out downstream differential methylation analyses using the package methylKit (Akalin et al., 2012) in R 4.0.4 (R Core Team, 2021). We merged strands for each CpG, and we only kept sites that had a depth of coverage of at least 10 reads for subsequent analyses. We further filtered out any site with a coverage higher than the 99.9th percentile of read counts. We deemed CpGs as methylated (hereafter, mCpGs) when their methylation level (i.e., the ratio of C to [C + T] reads at each CpG) was >10%. We defined differentially methylated cytosines (hereafter, DMCs) as CpGs with a methylation difference of 20% or greater between the two groups (i.e., conspecific‐cue exposed vs controls), a *q*‐value (Fisher's exact test corrected *p*‐value) (Wang et al., 2011) of 0.05 or less, and that were present in at least three samples in each group. We used the R package eDMR (Li et al., 2013) to identify differentially methylated regions (hereafter, DMRs). DMRs were defined as regions with a mean methylation difference of at least 20% between the two groups, a *q*‐value of 0.05 or less, and that contained at least 5 CpGs and 3 DMCs.

### 2.7 Effect of conspecific cues on tadpole growth

For each experiment, we investigated whether the exposure to conspecific cues influenced mass of focal tadpoles, using linear mixed‐effects models (LMMs) with the R package lme4 (Bates et al., 2015). We included mass as the dependent variable, treatment as a fixed effect, and clutch ID and replicate as random factors.

## 3 RESULTS

The average mass of tadpoles that had been exposed to cannibal cues as hatchlings was lower than controls in range‐edge populations, but not in range‐core populations (LMMs, respectively *p* < .00001 and *p* = .129; Figure 1b,c). Alarm cues had no significant effect on mean mass of exposed tadpoles in either range‐core or range‐edge populations (both *p* > .396; Figure 1d,e).

After filtering and quality trimming, the breadth of coverage (i.e., the number of CpGs with a coverage ≥10×) was 2.6 ± 0.1 million CpGs (mean ± SE) for tadpoles from the cannibal cue experiment, and 2.5 ± 0.1 million CpGs for tadpoles from the alarm cue experiment (Table A1 in Appendix). The depth of coverage was respectively 16.6 ± 0.1 and 16.8 ± 0.2 fold (Table A1 in Appendix).

**FIGURE 2:**
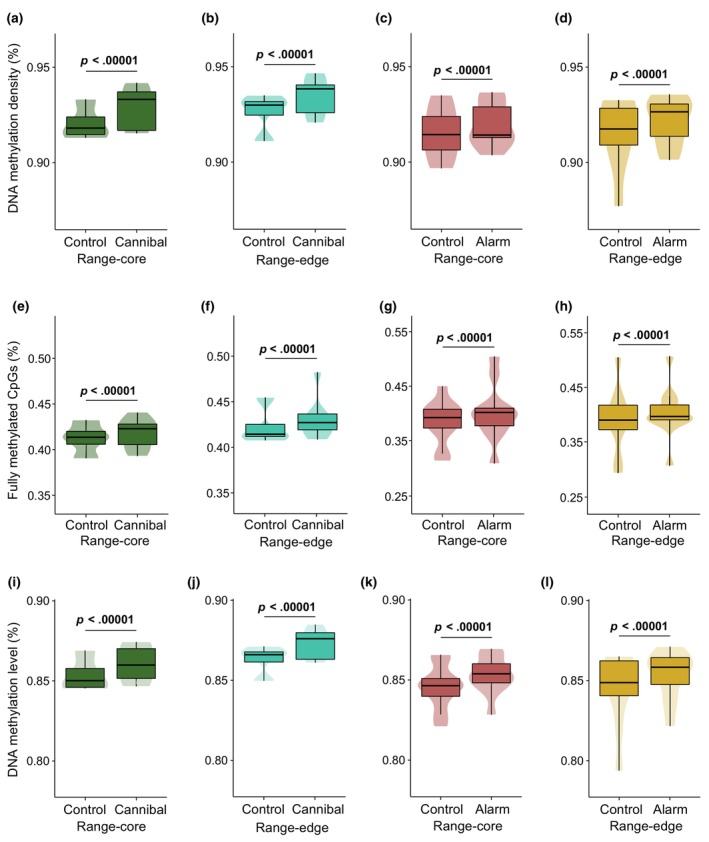
Patterns of DNA methylation in tadpoles exposed to conspecific cues and controls. (a–d) DNA methylation density (% of mCpGs out of all covered CpGs) in tadpoles exposed to cannibal cues and controls from (a) range‐core and (b) range‐edge populations, and in tadpoles exposed to alarm cues and controls from (c) range‐core and (d) range‐edge populations. (e–h) Proportion of fully methylated CpGs (%) in tadpoles exposed to cannibal cues and controls from (e) range‐core and (f) range‐edge populations, and in tadpoles exposed to alarm cues and controls from (g) range‐core and (h) range‐edge populations. (i–l) DNA methylation level (% of C out of [C + T] reads at each CpG) in tadpoles exposed to cannibal cues and controls from (i) range‐core and (j) range‐edge populations, and in tadpoles exposed to alarm cues and controls from (k) range‐core and (l) range‐edge populations. Violin plots represent median, interquartile range (IQR), 1.5 × IQR, and kernel density plot. Significant *p*‐values (GLMMs) are highlighted in bold.

The methylation density (i.e., the ratio of mCpGs to CpGs across the covered genome) was very high across all samples, as typically observed in vertebrates (Bird, 2002). Nonetheless, tadpoles exposed to either cannibal cues or alarm cues had higher methylation densities compared to controls in both range‐core and range‐edge populations (generalised linear mixed‐effects models [GLMMs], all *p* < .00001; Figure 2a–d). Likewise, the proportion of fully methylated sites was higher in tadpoles exposed to either cannibal cues or alarm cues compared to controls in both range‐core and range‐edge populations (GLMMs, all *p* < .00001; Figure 2e–h). Finally, the methylation level was also higher in tadpoles exposed to either cannibal cues or alarm cues compared to controls in both range‐core and range‐edge populations (GLMMs, all *p* < .00001; Figure 2i–l).

**FIGURE 3:**
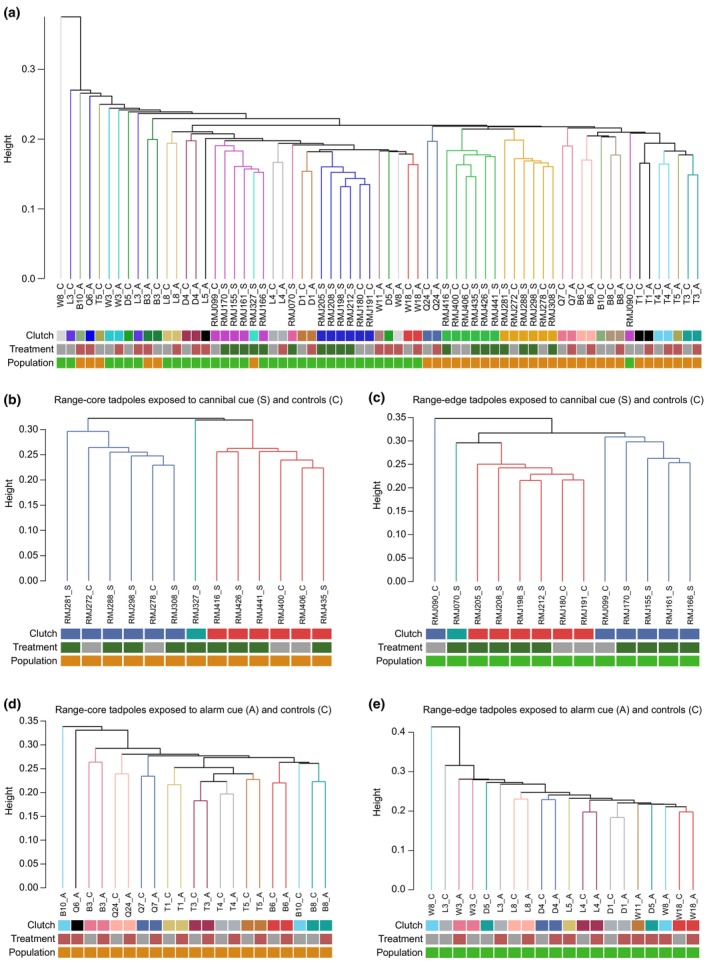
Hierarchical clustering (average agglomerative method on correlation distances) of samples according to their DNA methylation status. (a) All samples. (b, c) Samples exposed to cannibal cues and controls from (b) range‐core and (c) range‐edge populations. (d, e) Samples exposed to alarm cues and controls from (d) range‐core and (e) range‐edge populations. Coloured boxes group samples by clutch, treatment (i.e., red, alarm cue; dark green, cannibal cue; grey, control) and population (i.e., orange, range‐core; light green, range‐edge).

Hierarchical clustering based on methylation levels revealed a clear clustering by clutch identity for both range‐core and range‐edge populations (Figure 3). This pattern indicates that genetic differences were the main driver of epigenetic differences between samples, whereas conspecific cue exposure had a comparatively minor (albeit statistically significant) effect.

**FIGURE 4:**
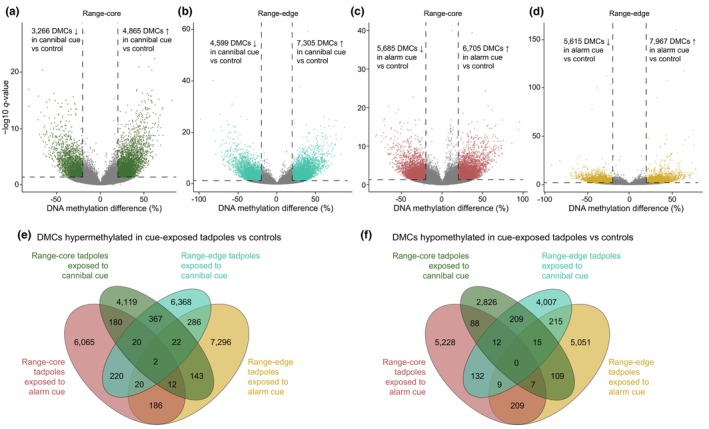
DNA methylation changes in tadpoles exposed to cannibal cues and alarm cues. (a–d) Volcano plots of DMCs between tadpoles exposed to cannibal cues and controls from (a) range‐core and (b) range‐edge populations, and between tadpoles exposed to alarm cues and controls from (c) range‐core and (d) range‐edge populations. Nonsignificant sites are represented in grey. (e, f) Venn diagrams represent the overlap of (e) hypermethylated and (f) hypomethylated DMCs in cannibal‐cue‐exposed tadpoles versus controls and in alarm‐cue‐exposed tadpoles versus controls, both from range‐core and range‐edge populations.

Across all groups, the number of DMCs in cue‐exposed tadpoles compared to controls ranged from 8131 to 13,582, of which 54.1%–61.4% were hypermethylated in tadpoles exposed to either cannibal cues or alarm cues (Figure 4a–d). Most DMCs (88.8% and 89.3% of hypermethylated and hypomethylated DMCs, respectively) were specific to tadpoles from one population exposed to one treatment (Figure 4e,f). Only 2 DMCs had higher methylation levels across all cue‐exposed tadpoles compared to controls, none of them intersecting with gene bodies (Figure 4e). No hypomethylated DMC was common across all groups (Figure 4f).

**FIGURE 5:**
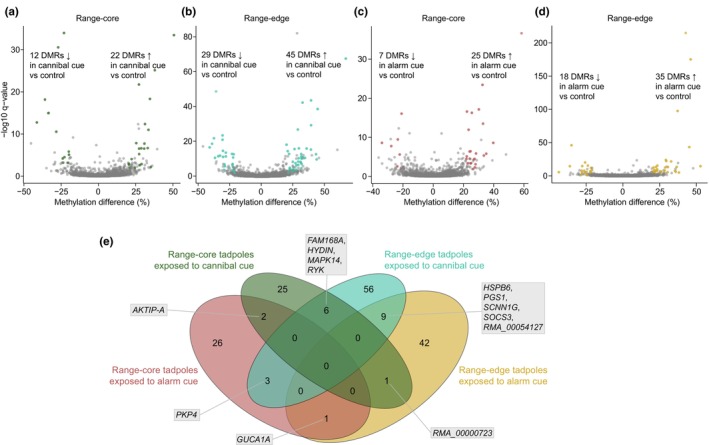
Local DNA methylation changes in tadpoles exposed to cannibal cues and alarm cues. (a–d) Volcano plots of DMRs between tadpoles exposed to cannibal cues and controls from (a) range‐core and (b) range‐edge populations, and between tadpoles exposed to alarm cues and controls from (c) range‐core and (d) range‐edge populations. Nonsignificant regions are represented in grey. (e) Venn diagram representing the overlap of DMRs in cannibal‐cue‐exposed tadpoles versus controls and in alarm‐cue‐exposed tadpoles versus controls, both from range‐core and range‐edge populations. Genes intersecting with overlapping DMRs are indicated.

There were 34 DMRs in range‐core tadpoles exposed to cannibal cues compared to controls, of which 20 (58.8%) were located within genes (Figure 5a and Table A2 in Appendix). In range‐edge tadpoles exposed to cannibal cues, there were 74 DMRs compared to controls, out of which 30 (40.5%) were found within genes, and 4 (5.4%) were located in promoter regions (Figure 5b and Table A3 in Appendix). DMRs each contained on average 5.4 ± 2.9 DMCs (mean ± SD; range 3–13) and 5.9 ± 3.0 DMCs (range 3–17) in range‐core and range‐edge tadpoles, respectively. The majority of DMRs were hypermethylated in cannibal‐cue‐exposed tadpoles compared to controls in both populations (respectively 64.7% and 60.8%; Figure 5a,b and Tables A2 and A3 in Appendix).

Only 6 DMRs overlapped across both populations in tadpoles exposed to cannibal cues compared to controls (Figure 5e), out of which four were located within genes *FAM168A*, *RYK*, *MAPK14*, and *HYDIN*. Further, only two overlapping DMRs (intersecting with *FAM168A* and *MAPK14*) showed parallel changes in DNA methylation levels in both populations, while the other four showed opposite changes in range‐core and range‐edge tadpoles compared to controls.

There were 32 DMRs between range‐core tadpoles exposed to alarm cues and controls, out of which 14 (43.8%) were found within genic regions, and 3 (9.4%) were found in promoter regions (Figure 5c and Table A4 in Appendix). In range‐edge tadpoles exposed to alarm cues, there were 53 DMRs compared to controls, out of which 27 (50.9%) were located within genes, and 1 (1.9%) was found in promoter regions (Figure 5d and Table A5 in Appendix). Each DMR contained on average 4.6 ± 2.1 DMCs (range 3–12) in range‐core tadpoles, and 6.6 ± 4.2 DMCs (range 3–25) in range‐edge tadpoles. As for cannibal‐cue‐exposed tadpoles, most DMRs showed a significant increase in DNA methylation level compared to controls in both range‐core and range‐edge populations (respectively 78.1% and 66.0%; Figure 5c,d and Tables A4 and A5 in Appendix).

Only 1 DMR, located within the *GUCA1A* gene, overlapped and showed an increase in DNA methylation levels across both populations in tadpoles exposed to alarm cues compared to controls (Figure 5e).

Overall, there was minimal overlap in regions showing differential methylation in tadpoles exposed to cannibal cues and in tadpoles exposed to alarm cues compared to controls (Figure 5e). Only 2 DMRs overlapped between cannibal‐cue‐exposed and alarm‐cue‐exposed tadpoles from range‐core populations. These DMRs were located respectively in the *AKTIP‐A* gene, and in an intergenic region. The first DMR showed opposite changes in DNA methylation levels in both treatments relative to controls, while the second one showed hypermethylation in both treatments relative to controls. In range‐edge tadpoles, 9 DMRs overlapped between tadpoles exposed to cannibal cues and those exposed to alarm cues. Four of those DMRs intersected with genes *HSPB6*, *PGS1*, *SCNN1G*, *SOCS3*, as well as the uncharacterised transcript *RMA_00054127*. However, only genes *PGS1*, *SCNN1G* and *SOCS3* exhibited parallel changes in DNA methylation levels in both treatments relative to controls (hypomethylation for *PGS1* and *SOCS3* and hypermethylation for *SCNN1G*). Of the 5 remaining intergenic DMRs, 3 showed parallel changes and 2 showed opposite changes in DNA methylation levels in both treatments relative to controls.

One DMR (showing opposite changes in DNA methylation levels and intersecting with uncharacterised transcript *RMA_00000723*) overlapped between range‐core tadpoles exposed to cannibal cues and range‐edge tadpoles exposed to alarm cues. Lastly, three DMRs overlapped between range‐edge tadpoles exposed to cannibal cues and range‐core tadpoles exposed to alarm cues. Only one of those DMRs was located within gene *PKP4* and showed opposite changes in DNA methylation levels in both treatments relative to controls.

There was no DMR overlap across both treatments and both populations (Figure 5e). GO enrichment analyses did not reveal any significantly over‐represented GO term for all DMR lists.

## 4 DISCUSSION

Our findings reveal that exposure to conspecific cannibal cues and alarm cues were both associated with changes in the DNA methylation profiles of cane toad tadpoles, but in different ways. Our results thus suggest that the developmental plastic responses seen in these two contexts, despite their similar short‐ and long‐term consequences, are underpinned by distinct molecular mechanisms.

We did observe a similar overall pattern of hypermethylation in tadpoles exposed to both conspecific cues in both populations compared to controls. These slightly higher levels of DNA methylation might indicate marginally lower levels of gene expression (Jaenisch & Bird, 2003) in tadpoles exposed to conspecific cues. Nonetheless, these changes were small (typically <1%), and the relationship between DNA methylation levels and transcriptional activity are far from being universal and unidirectional (de Mendoza et al., 2020).

We also observed some overlap in DMRs between cannibal‐cue‐exposed tadpoles compared to controls and alarm‐cue‐exposed tadpoles compared to controls. Two DMRs overlapped between both treatments in range‐core tadpoles, and 9 DMRs overlapped between both treatments in range‐edge tadpoles. These DMRs were found within the gene bodies of *AKTIP‐A*, *HSPB6*, *PGS1*, *SCNN1G*, *SOCS3*, as well as two uncharacterised genes. *AKTIP‐A* mouse mutants show developmental abnormalities (Anselme et al., 2007). *HSPB6* is involved in the regulation of angiogenesis (Vafiadaki et al., 2020), *SCNN1G* plays a role in water homeostasis (Hobbs et al., 2013), while *SOCS3* is involved in the regulation of food intake (Zhu et al., 2021). DNA methylation patterns of 5′‐flanking regions have been shown to correlate with gene expression, at least for *SCNN1G* (Pierandrei et al., 2021). Changes in DNA methylation in these genes were already evidenced in cane toad tadpoles following exposure to alarm cues (Sarma et al., 2020, 2021), and we here show that the same modifications occur after exposure to cannibal cues. These findings offer a possible mechanistic link explaining why individuals exposed to alarm cues produce offspring that emit more potent cannibal cues (Sarma et al., 2021).

Changes in DNA methylation levels in the above‐mentioned genes may have developmental consequences during cane toads' larval life. Yet, as well as being restricted to only a few overlapping regions, changes in DNA methylation levels were not always consistent across treatments (e.g., hypermethylation in one case and hypomethylation in the other). This casts doubts on the causative link between changes in DNA methylation levels, changes in gene expression levels, and downstream phenotypic consequences due to exposure to cannibal cues and alarm cues. The overlap in DMCs was also minimal, and it is unclear mechanistically how changes in DNA methylation levels of single CpGs can affect gene activity (but see e.g., Sobiak & Leśniak, 2019).

Within each treatment, additional DMRs intersected with genes also putatively involved in developmental processes. For example, cannibal‐cue‐exposed tadpoles showed differences in DNA methylation levels in the genes *HDYN*, *LARGE1*, *LRP4*, *MAPK14*, *NIN*, *NLK*, *PLXNA1*, *RANBP3L*, *RYK*, *SEZ6* and *SOX5*. *HDYN*, *PLXNA1* and SOX5 play a role in brain development (Andrews et al., 2016; Palmer et al., 2016; Stolt et al., 2006). *LARGE1*, *MAPK14*, *NLK*, *RANBP3L* and *RYK* are involved in skeletal system development (Chen et al., 2015; Goddeeris et al., 2013; Greenblatt et al., 2010; Halford et al., 2000; Zanotti & Canalis, 2012). *LRP4* plays a role in normal organ development (Weatherbee et al., 2006). *NIN* is integral to epidermis development (Lecland et al., 2019). *SEZ6* is involved in the regulation of motor functions (Gunnersen et al., 2020). Alarm‐cue‐exposed tadpoles showed differences in DNA methylation levels in the genes *CEP135*, *DACH2*, *ECE1*, *EIF3A*, *ENG*, *GPR155*, *MINK1*, *MYL3*, *PKD1*, *TIE1* and *ZCCHC3*. *CEP135* and *MINK1* are involved in brain development (Bamborschke et al., 2020; Dan et al., 2000). *DACH2* plays a role in muscle development (Tang & Goldman, 2006). *ECE1*, *ENG*, *MYL3* and *PKD1* are involved in heart development (Arthur et al., 2000; Boulter et al., 2001; James et al., 1999; Poltavski et al., 2019). *EIF3A* plays a role in intestinal development (Liu et al., 2007). *TIE1* is involved in angiogenesis (Loughna & Sato, 2001). *ZCCHC3* plays a role in innate immune response (Lian et al., 2018). Finally, *GPR155* is involved in cognitive functions (Nishimura et al., 2007). Overall, these genes are interesting candidates for future studies, whose main focus should be directed towards generating gene expression data for tadpoles exposed to both conspecific cues and controls across development. This should help to confirm that the above‐mentioned genes that show differences in DNA methylation levels between treatments also show differences in gene expression levels, and should bring us one step closer to establishing a causal link between molecular mechanisms and developmental plasticity.

Our results revealed clearly that epigenetic differences between tadpoles were mostly driven by their clutch identity. This phenomenon has previously been documented in cane toads (Sarma et al., 2020), and indicates that genetic differences between individuals are the main cause for their divergence in DNA methylation patterns. The influence of genotypic variation on DNA methylation marks appears ubiquitous. Mounting evidence shows that, although epigenetic marks can be modified by environmental exposure, in many cases they do so under genetic control (Do et al., 2017; Gaunt et al., 2016; Hannon et al., 2018; Kerkel et al., 2008; Min et al., 2021; Tycko, 2010; Villicaña & Bell, 2021). These results stress the importance of controlling for genetic effects (i.e., having a balanced experimental design in terms of clutch identity) when investigating differences in DNA methylation patterns between treatment and control. These results also help explain why we observed only minimal overlap in DMRs between range‐core and range‐edge tadpoles, even within each treatment (i.e., for tadpoles exposed to the same conspecific cue). Because tadpoles from distinct populations necessarily came from distinct clutches, their constitutive genetic differences induced population‐specific DNA methylation patterns (prior to any conspecific cue exposure) that were of greater magnitude than the effect of treatment itself. Our findings further complement previous results showing that clutches vary in their reaction norms, i.e., in their propensity to accelerate their development, when exposed to conspecific cues (DeVore, Crossland, & Shine, 2021).

We found that changes in DNA methylation following exposure to both cannibal cues and alarm cues were largely population‐specific, and were of greater magnitude in range‐edge tadpoles than in range‐core tadpoles. This effect was mirrored in the developmental effects of conspecific cue exposure (i.e., greater effect on mass at the range‐edge). These population‐specific DNA methylation patterns are consistent with previous studies investigating epigenetic patterns in cane toads (Sarma et al., 2020, 2021). More generally, they are consistent with the well documented between‐population differences in cane toad morphology (Hudson et al., 2016; Phillips et al., 2006), physiology (Brown et al., 2015), behaviour (Gruber et al., 2017; Lindstrom et al., 2013), transcriptomics (Rollins et al., 2015; Yagound, West, Richardson, Gruber, et al., 2022; Yagound, West, Richardson, Selechnik, et al., 2022) and genetics (Selechnik et al., 2019) across the Australian invasive range.

We did not detect any significant reduction in body mass in alarm‐cue‐exposed tadpoles. By contrast, previous studies found such an effect at a later stage in development (i.e., at metamorphosis) (Crossland et al., 2019; Hagman et al., 2009; Hagman & Shine, 2009). Thus, the apparent lack of growth reduction seen following alarm‐cue exposure may be an artefact of early euthanasia.

Exposure to cannibal cues and alarm cues thus appear to be associated with distinct molecular mechanisms. Both cues are correlated with changes in DNA methylation patterns locally, but each in largely distinct genomic regions. It is interesting to contrast these findings with the observations that both cues trigger reduced growth responses in tadpoles (Crossland & Shine, 2012; DeVore, Crossland, & Shine, 2021; Hagman et al., 2009; Hagman & Shine, 2009), and that hatchlings exposed to alarm cues have offspring that themselves produce more potent cannibal cues (Sarma et al., 2021). Several hypotheses might explain this discrepancy. Each cue might trigger a series of molecular changes involving many genes within complex networks. It is possible that each cue does indeed involve distinct causal molecular mechanisms that result in similar phenotypic effects. While growth reduction in tadpoles is a direct consequence in the case of exposure to alarm cues (Crossland et al., 2019; Hagman et al., 2009; Hagman & Shine, 2009), it is a carry‐over effect of the hatchling stage in the case of exposure to cannibal cues (Clarke et al., 2015; Crossland & Shine, 2012; DeVore, Crossland, & Shine, 2021), which might contribute to the lack of overlap in DNA methylation changes seen between exposure to both conspecific cues. By contrast, it is also possible that these gene networks are quite similar between both contexts, but that we were only able to capture a fraction of the genomic regions involved in each case. The lack of overlap could then derive from constraints in our experimental design in terms of sample size, statistical power, sequencing methodology, breadth of coverage and/or underlying genetic differences. If the molecular changes underlying developmental plasticity are restricted to a short time‐window, it is possible that we sampled tadpoles too late to detect them. Our experiments were conducted at different times, and involved tadpoles of slightly different ages, which could also have introduced artefacts in our results. Lastly, is it also possible that the changes we observed in DNA methylation patterns are not causally involved in cane toad developmental plasticity. DNA methylation marks might well be affected by exposure to conspecific cues, but perhaps these changes are by‐products of conspecific‐cue exposure, or a consequence of other molecular changes (perhaps also epigenetic in nature, such as histone post‐translational modifications; Cedar & Bergman, 2009) that are themselves the cause of downstream developmental changes. Gene expression data matched to epigenetic data are needed to solve this enduring puzzle.

## AUTHOR CONTRIBUTIONS

**Boris Yagound:** Data curation (lead); formal analysis (lead); writing – original draft (lead); writing – review and editing (lead). **Roshmi R. Sarma:** Formal analysis (equal); investigation (lead); methodology (equal); resources (equal); writing – review and editing (supporting). **Richard J. Edwards:** Data curation (equal); formal analysis (equal); methodology (equal); resources (equal); software (equal). **Mark F. Richardson:** Conceptualization (equal); methodology (equal); resources (equal). **Carlos M. Rodriguez Lopez:** Conceptualization (equal); methodology (equal); resources (equal); supervision (supporting); writing – review and editing (equal). **Michael R. Crossland:** Investigation (equal); methodology (equal); resources (equal); supervision (equal); writing – review and editing (equal). **Gregory P. Brown:** Data curation (equal); formal analysis (equal); investigation (equal); methodology (equal); resources (equal); supervision (equal); visualization (supporting). **Jayna L. DeVore:** Conceptualization (equal); methodology (equal); resources (equal); writing – review and editing (equal). **Richard Shine:** Conceptualization (equal); funding acquisition (lead); methodology (equal); project administration (equal); resources (equal); supervision (supporting); writing – review and editing (equal). **Lee A. Rollins:** Conceptualization (lead); funding acquisition (lead); investigation (equal); methodology (equal); project administration (lead); resources (equal); supervision (lead); writing – review and editing (lead).

## FUNDING INFORMATION

This work was supported by a Deakin University Fellowship and a UNSW Scientia Fellowship to LAR, and the Australian Research Council (DE150101393 to LAR, FL120100074 to RS, and DP160102991 to RS and LAR).

## CONFLICT OF INTEREST STATEMENT

The authors declare no conflicts of interest.

## Data Availability

The RRBS data for the 26 samples from the cannibal cue experiment, and the 41 samples from the alarm cue experiment is available at the National Center for Biotechnology Information (NCBI) Sequence Read Archive (BioProject PRJNA901184).

## Appendix

**TABLE A1:** DNA methylation statistics for each sample.

Individual	Population	Treatment	Coverage (mean ± SE)	No. covered CpGs (>10×)	No. fully methylated CpGs (%)	DNA methylation level (%, mean ± SE)
B10_C	Range‐core	Control	17.11 ± 0.01	2,706,712	1,012,241 (37.40)	85.19 ± 0.01
B10_A	Range‐core	Alarm cue	15.82 ± 0.01	1,851,760	870,936 (47.03)	84.49 ± 0.02
B3_C	Range‐core	Control	16.07 ± 0.01	2,120,158	956,668 (45.12)	84.52 ± 0.02
B3_A	Range‐core	Alarm cue	17.63 ± 0.01	2,882,616	1,198,234 (41.57)	86.43 ± 0.01
B6_C	Range‐core	Control	17.08 ± 0.01	2,685,849	1,099,764 (40.95)	86.34 ± 0.01
B6_A	Range‐core	Alarm cue	19.01 ± 0.01	3,466,836	1,396,244 (40.27)	86.30 ± 0.01
B8_C	Range‐core	Control	16.82 ± 0.01	2,654,890	989,638 (37.28)	84.77 ± 0.01
B8_A	Range‐core	Alarm cue	16.45 ± 0.01	2,597,621	996,799 (38.37)	84.89 ± 0.02
Q24_C	Range‐core	Control	16.27 ± 0.01	2,277,887	943,133 (41.40)	84.47 ± 0.02
Q24_A	Range‐core	Alarm cue	17.21 ± 0.01	2,377,544	955,539 (40.19)	85.90 ± 0.01
Q6_A	Range‐core	Alarm cue	16.01 ± 0.01	1,897,238	952,975 (50.23)	84.99 ± 0.02
Q7_C	Range‐core	Control	15.66 ± 0.01	2,062,043	834,106 (40.45)	83.82 ± 0.02
Q7_A	Range‐core	Alarm cue	16.30 ± 0.01	2,500,833	1,009,438 (40.36)	84.44 ± 0.02
T1_C	Range‐core	Control	18.49 ± 0.01	2,854,455	1,152,233 (40.37)	86.57 ± 0.01
T1_A	Range‐core	Alarm cue	16.82 ± 0.01	2,911,986	1,139,738 (39.14)	85.13 ± 0.01
T3_C	Range‐core	Control	17.07 ± 0.01	2,704,868	845,071 (31.24)	82.12 ± 0.02
T3_A	Range‐core	Alarm cue	16.99 ± 0.01	3,025,076	1,087,944 (35.96)	83.83 ± 0.02
T4_C	Range‐core	Control	16.91 ± 0.01	3,188,903	1,216,855 (38.16)	84.82 ± 0.01
T4_A	Range‐core	Alarm cue	16.94 ± 0.01	2,712,431	842,394 (31.06)	82.37 ± 0.02
T5_C	Range‐core	Control	17.95 ± 0.01	2,149,651	699,234 (32.53)	82.87 ± 0.02
T5_A	Range‐core	Alarm cue	16.04 ± 0.01	2,820,292	1,050,226 (37.24)	84.23 ± 0.02
D1_C	Range‐edge	Control	17.76 ± 0.01	3,379,797	1,255,520 (37.15)	85.42 ± 0.01
D1_A	Range‐edge	Alarm cue	18.33 ± 0.01	3,503,050	1,367,471 (39.04)	86.13 ± 0.01
D4_C	Range‐edge	Control	17.46 ± 0.01	2,711,875	1,126,971 (41.56)	86.28 ± 0.01
D4_A	Range‐edge	Alarm cue	16.46 ± 0.01	2,493,594	765,879 (30.71)	81.99 ± 0.02
D5_C	Range‐edge	Control	15.31 ± 0.01	1,606,267	472,261 (29.40)	79.22 ± 0.02
D5_A	Range‐edge	Alarm cue	16.84 ± 0.01	2,979,308	1,155,748 (38.79)	85.62 ± 0.01
L3_C	Range‐edge	Control	16.13 ± 0.01	1,772,721	888,587 (50.13)	84.67 ± 0.02
L3_A	Range‐edge	Alarm cue	15.29 ± 0.01	1,688,226	668,014 (39.57)	83.29 ± 0.02
L4_C	Range‐edge	Control	16.52 ± 0.01	2,408,495	759,380 (31.53)	81.88 ± 0.02
L4_A	Range‐edge	Alarm cue	16.93 ± 0.01	3,183,418	1,242,975 (39.05)	85.67 ± 0.01
L5_A	Range‐edge	Alarm cue	17.68 ± 0.01	2,717,554	1,104,151 (40.63)	86.31 ± 0.01
L8_C	Range‐edge	Control	15.65 ± 0.01	2,213,397	860,310 (38.87)	83.86 ± 0.02
L8_A	Range‐edge	Alarm cue	15.39 ± 0.01	2,035,356	804,367 (39.52)	83.95 ± 0.02
W11_A	Range‐edge	Alarm cue	16.63 ± 0.01	2,561,577	1,105,457 (43.16)	86.36 ± 0.01
W18_C	Range‐edge	Control	17.49 ± 0.01	3,559,635	1,380,915 (38.79)	86.02 ± 0.01
W18_A	Range‐edge	Alarm cue	17.22 ± 0.01	2,638,045	1,122,813 (42.56)	86.88 ± 0.01
W3_C	Range‐edge	Control	15.61 ± 0.01	1,268,902	547,030 (43.11)	86.14 ± 0.02
W3_A	Range‐edge	Alarm cue	16.75 ± 0.01	2,395,814	1,205,656 (50.32)	85.37 ± 0.02
W8_C	Range‐edge	Control	18.27 ± 0.03	676,604	268,837 (39.73)	84.33 ± 0.03
W8_A	Range‐edge	Alarm cue	16.50 ± 0.01	3,059,678	1,175,334 (38.41)	85.16 ± 0.01
RMJ272_C	Range‐core	Control	15.41 ± 0.01	2,023,970	840,345 (41.52)	84.89 ± 0.02
RMJ278_C	Range‐core	Control	16.07 ± 0.01	2,578,716	1,057,554 (41.01)	85.67 ± 0.02
RMJ281_S	Range‐core	Cannibal cue	16.30 ± 0.01	2,114,801	930,636 (44.01)	85.07 ± 0.02
RMJ288_S	Range‐core	Cannibal cue	15.93 ± 0.01	2,377,902	948,244 (39.88)	84.93 ± 0.02
RMJ298_S	Range‐core	Cannibal cue	15.84 ± 0.01	2,246,608	948,199 (42.21)	85.42 ± 0.02
RMJ308_S	Range‐core	Cannibal cue	17.11 ± 0.01	2,761,054	1,170,417 (42.39)	87.17 ± 0.01
RMJ327_S	Range‐core	Cannibal cue	17.24 ± 0.01	3,267,404	1,405,491 (43.02)	87.68 ± 0.01
RMJ400_C	Range‐core	Control	16.95 ± 0.01	2,696,471	1,163,267 (43.14)	87.14 ± 0.01
RMJ406_C	Range‐core	Control	16.29 ± 0.01	2,641,992	1,028,735 (38.94)	84.79 ± 0.02
RMJ416_S	Range‐core	Cannibal cue	17.40 ± 0.01	2,280,409	974,285 (42.72)	87.50 ± 0.01
RMJ426_S	Range‐core	Cannibal cue	17.44 ± 0.01	2,760,301	1,082,488 (39.22)	86.25 ± 0.01
RMJ435_S	Range‐core	Cannibal cue	16.48 ± 0.01	2,454,708	993,000 (40.45)	85.69 ± 0.02
RMJ441_S	Range‐core	Cannibal cue	18.35 ± 0.01	2,884,432	1,192,090 (41.33)	87.27 ± 0.01
RMJ070_S	Range‐edge	Cannibal cue	16.96 ± 0.01	2,452,473	1,067,309 (43.52)	88.06 ± 0.01
RMJ090_C	Range‐edge	Control	15.72 ± 0.01	2,365,636	975,369 (41.23)	84.59 ± 0.02
RMJ099_C	Range‐edge	Control	16.19 ± 0.01	2,410,288	1,088,735 (45.17)	86.14 ± 0.02
RMJ155_S	Range‐edge	Cannibal cue	16.26 ± 0.01	2,449,011	1,062,206 (43.37)	85.90 ± 0.02
RMJ161_S	Range‐edge	Cannibal cue	16.08 ± 0.01	2,714,595	1,101,465 (40.58)	85.73 ± 0.01
RMJ166_S	Range‐edge	Cannibal cue	16.28 ± 0.01	2,898,435	1,206,304 (41.62)	85.92 ± 0.01
RMJ170_S	Range‐edge	Cannibal cue	16.61 ± 0.01	2,583,042	1,239,096 (47.97)	86.31 ± 0.01
RMJ180_C	Range‐edge	Control	16.38 ± 0.01	2,853,992	1,170,950 (41.03)	86.70 ± 0.01
RMJ191_C	Range‐edge	Control	16.45 ± 0.01	3,063,958	1,239,234 (40.45)	86.27 ± 0.01
RMJ198_S	Range‐edge	Cannibal cue	17.37 ± 0.01	3,081,851	1,284,695 (41.69)	87.20 ± 0.01
RMJ205_S	Range‐edge	Cannibal cue	17.52 ± 0.01	2,671,839	1,093,008 (40.91)	87.62 ± 0.01
RMJ208_S	Range‐edge	Cannibal cue	16.54 ± 0.01	2,653,964	1,125,590 (42.41)	87.20 ± 0.01
RMJ212_S	Range‐edge	Cannibal cue	17.38 ± 0.01	2,990,400	1,270,081 (42.47)	87.57 ± 0.01

**TABLE A2:** DMRs between range‐core tadpoles exposed to cannibal cues and controls.

Contig	Start DMR	End DMR	Length DMR (bp)	Methylation difference (%)	No. CpGs	No. DMCs	DMR	Gene	Protein
							*q*‐Value		
ctg7929	70,227	70,401	175	−41.55	9	8	<0.00001	*RBKS*	Ribokinase
ctg399^b^	944,763	945,066	304	−36.11	15	11	<0.00001	*RMA_00000723*	Unknown
ctg6739	25,009	25,071	63	−33.86	6	6	<0.00001	Intergenic	N/A
ctg3195^a^	95,407	95,827	421	−28.54	15	8	<0.00001	*AKTIP‐A*	AKT‐interacting protein homolog A
ctg11181	114,196	114,447	252	−27.27	10	8	<0.00001	Intergenic	N/A
ctg3933	41,095	41,188	94	−24.24	8	4	0.00001	*TXN*	Thioredoxin
ctg14726^c^	68,308	68,398	91	−24.17	6	3	0.00013	*RYK*	Tyrosine‐protein kinase RYK
ctg15338	47,218	47,513	296	−23.37	13	13	<0.00001	*SLC47A1*	Multidrug and toxin extrusion protein 1
ctg3167^c^	901,657	902,160	504	−23.06	12	5	<0.00001	Intergenic	N/A
ctg23866	3072	3162	91	−20.59	8	3	0.00013	Intergenic	N/A
ctg4387	339,637	339,733	97	−20.16	6	3	<0.00001	Intergenic	N/A
ctg12090	137,482	137,780	299	−20.01	7	4	<0.00001	*SEZ6*	Seizure protein 6 homolog
ctg770	188,230	188,323	94	20.05	8	3	0.00021	*PDE1A*	Calcium/calmodulin‐dependent 3′,5′‐cyclic nucleotide phosphodiesterase 1A
ctg998^c^	34,962	35,753	792	20.07	19	3	0.00033	*HYDIN*	Hydrocephalus‐inducing protein homolog
ctg21927	490	537	48	20.69	9	4	0.0023	Intergenic	N/A
ctg4046	135,831	136,086	256	21.93	14	4	0.00002	Intergenic	N/A
ctg3168	255,027	255,218	192	24.09	8	4	0.0022	*SHANK3*	SH3 and multiple ankyrin repeat domains protein 3
ctg76	446,909	447,163	255	25.04	12	4	<0.00001	*TMEM163*	Transmembrane protein 163
ctg3843	40,389	40,436	48	26.77	5	3	<0.00001	Intergenic	N/A
ctg29953	24,973	25,187	215	26.82	8	6	<0.00001	Intergenic	N/A
ctg143	70,476	70,895	420	26.94	16	10	<0.00001	*RMA_00025525*	Unknown
ctg7863^c^	136,579	136,654	76	26.97	5	3	<0.00001	Intergenic	N/A
ctg4646	112,284	112,345	62	27.46	5	3	0.00046	*NIN*	Ninein
ctg1638^c^	23,284	23,587	304	28.42	9	7	<0.00001	*MAPK14*	Mitogen‐activated protein kinase 14
ctg25158	53,008	53,098	91	28.58	5	3	0.00026	Intergenic	N/A
ctg6921	20,708	20,779	72	30.34	7	6	<0.00001	*SMIM4*	Small integral membrane protein 4
ctg11248	90,859	90,933	75	30.51	6	4	<0.00001	Intergenic	N/A
ctg29669	10,257	10,358	102	30.89	7	5	<0.00001	Intergenic	N/A
ctg9969^a^	28,668	28,807	140	32.70	5	4	<0.00001	Intergenic	N/A
ctg3847	231,861	231,939	79	33.17	5	3	<0.00001	*VSTM2B*	V‐set and transmembrane domain‐containing protein 2B
ctg816	330,047	330,389	343	34.19	13	11	<0.00001	*RMA_00012239*	Unknown
ctg865	445,930	446,083	154	34.83	5	3	0.0012	*RMA_00005388*	Unknown
ctg7585^c^	41,611	41,764	154	37.45	12	11	<0.00001	*FAM168A*	Protein FAM168A
ctg4558	49,492	49,577	86	50.27	5	5	<0.00001	*ADAMTS14*	A disintegrin and metalloproteinase with thrombospondin motifs 14

^a^ Also found between range‐core tadpoles exposed to alarm cues and controls.

^b^ Also found between range‐edge tadpoles exposed to alarm cues and controls.

^c^ Also found between range‐edge tadpoles exposed to cannibal cues and controls.

**TABLE A3:** DMRs between range‐edge tadpoles exposed to cannibal cues and controls.

Contig	Start DMR	End DMR	Length DMR (bp)	Methylation difference (%)	No. CpGs	No. DMCs	DMR	Gene	Protein
							*q*‐Value		
ctg1071	247,181	247,373	193	−35.69	18	17	<0.00001	*SOX5*	Transcription factor SOX‐5
ctg1072	44,611	44,778	168	31.24	7	4	<0.00001	*RANBP3L*	Ran‐binding protein 3‐like
ctg1164	140,743	140,808	66	−22.74	8	4	0.0011	Intergenic	N/A
ctg1181^b^	2961	3336	376	−33.97	15	9	<0.00001	Intergenic	N/A
ctg12002	173,499	173,586	88	−30.54	8	8	<0.00001	*RMA_00019178*	Unknown
ctg12558	33,641	34,312	672	21.03	15	7	<0.00001	*APEH*	Acylamino‐acid‐releasing enzyme
ctg12829	58,878	58,963	86	32.94	7	7	<0.00001	Intergenic	N/A
ctg13603	25,150	25,214	65	29.89	6	6	<0.00001	Promoter of *OAZ1*	Ornithine decarboxylase antizyme 1
ctg13974	11,408	11,564	157	−21.75	8	6	<0.00001	Promoter of *GLRA2*	Glycine receptor subunit alpha‐2
ctg14020	10,370	10,474	105	−30.25	7	6	<0.00001	Intergenic	N/A
ctg14606	9914	10,042	129	−28.66	7	5	<0.00001	Intergenic	N/A
ctg14726^c^	68,334	68,398	65	25.06	5	4	<0.00001	*RYK*	Tyrosine‐protein kinase RYK
ctg14814	121,525	121,602	78	32.10	5	3	<0.00001	Intergenic	N/A
ctg15409	4923	5040	118	20.13	7	6	<0.00001	Promoter of *RMA_00024538*	Unknown
ctg1590	79,976	80,019	44	43.91	5	5	<0.00001	*LARGE1*	LARGE xylosyl‐ and glucuronyltransferase 1
ctg16265	35,720	35,795	76	34.76	6	5	<0.00001	*LRP4*	Low‐density lipoprotein receptor‐related protein 4
ctg1638^c^	23,227	23,361	135	32.19	7	6	<0.00001	*MAPK14*	Mitogen‐activated protein kinase 14
ctg1846	86,117	86,265	149	−25.13	5	3	<0.00001	*FOLH1*	Glutamate carboxypeptidase 2
ctg189	6300	6432	133	22.69	8	3	0.00023	Intergenic	N/A
ctg19321^b^	2398	2462	65	−22.11	6	3	0.00040	*RMA_00054127*	Unknown
ctg19786	63,399	63,623	225	22.61	12	5	0.00001	Intergenic	N/A
ctg20065	18,387	18,461	75	25.88	5	3	<0.00001	Intergenic	N/A
ctg2037	59,449	59,536	88	−36.60	9	7	<0.00001	Intergenic	N/A
ctg2041	250,592	250,698	107	28.26	6	4	0.00009	Intergenic	N/A
ctg21629	33,580	33,848	269	27.94	6	4	<0.00001	Intergenic	N/A
ctg2173	117,678	117,812	135	−30.16	8	5	<0.00001	*KNL1*	Kinetochore scaffold 1
ctg22578	73,170	73,239	70	21.12	5	3	0.0035	*RMA_00027688*	Unknown
ctg22943	7589	8105	517	−22.20	16	7	<0.00001	Intergenic	N/A
ctg2436	4131	4196	66	−32.54	5	4	<0.00001	*L1RE1*	LINE‐1 retrotransposable element ORF2 protein
ctg25051	4062	4376	315	29.09	21	14	<0.00001	Intergenic	N/A
ctg25970^b^	30,960	31,112	153	39.54	13	13	<0.00001	Intergenic	N/A
ctg26570	11,352	11,400	49	−29.03	5	4	<0.00001	Intergenic	N/A
ctg2710	697,395	698,125	731	−23.09	28	11	<0.00001	*RPTOR*	Regulatory‐associated protein of mTOR
ctg2897	819,247	819,342	96	44.75	7	7	<0.00001	Intergenic	N/A
ctg29626	14,134	14,606	473	22.17	12	10	<0.00001	Intergenic	N/A
ctg3167^c^	901,657	902,160	504	31.69	12	7	<0.00001	Intergenic	N/A
ctg3234	10,302	10,388	87	31.86	8	3	<0.00001	Intergenic	N/A
ctg3326	266,723	266,774	52	26.53	6	3	0.00010	*PLXNA1*	Plexin‐A1
ctg3451	4391	4486	96	39.43	5	4	<0.00001	Intergenic	N/A
ctg3568	716,102	716,174	73	29.71	5	5	<0.00001	Intergenic	N/A
ctg3738	186,066	186,201	136	27.92	6	3	0.00003	*MMCM3*	Maternal DNA replication licensing factor mcm3
ctg3758^b^	44,460	44,677	218	−27.97	13	9	<0.00001	Intergenic	N/A
ctg3759	6756	6870	115	−38.87	7	7	<0.00001	Intergenic	N/A
ctg3773^b^	120,249	120,346	98	−22.77	7	5	<0.00001	Intergenic	N/A
ctg3773^b^	120,787	121,112	326	−22.58	22	10	<0.00001	*SOCS3*	Suppressor of cytokine signaling 3
ctg3773^b^	171,406	171,542	137	−22.15	10	5	0.00004	*PGS1*	CDP‐diacylglycerol‐glycerol‐3‐phosphate 3‐phosphatidyltransferase, mitochondrial
ctg4046	115,513	115,634	122	26.41	6	4	<0.00001	*RAB6A*	Ras‐related protein Rab‐6A
ctg42^a^	1,763,592	1,763,650	59	31.94	6	6	<0.00001	*PKP4*	Plakophilin‐4
ctg4628	112,210	112,354	145	22.45	13	4	0.00028	Intergenic	N/A
ctg4676	16,488	16,607	120	−21.78	7	3	<0.00001	*TCB1*	Transposable element Tcb1 transposase
ctg4820	179,259	179,361	103	22.70	9	4	<0.00001	*SKIV2L2*	Superkiller viralicidic activity 2‐like 2
ctg5009	155,256	155,336	81	31.43	6	5	<0.00001	Intergenic	N/A
ctg505	18,210	18,349	140	−37.37	8	8	<0.00001	Intergenic	N/A
ctg508	90,532	90,621	90	22.70	11	5	<0.00001	Promoter of *RMA_00023488*	Unknown
ctg5287	289,785	289,864	80	−27.96	10	8	<0.00001	*TRMT61A*	tRNA (adenine(58)‐N(1))‐methyltransferase catalytic subunit TRMT61A
ctg5424^b^	170,718	170,848	131	36.03	5	4	<0.00001	*HSPB6*	Heat shock protein beta‐6
ctg6018	72,530	72,774	245	25.33	8	3	0.00005	Intergenic	N/A
ctg6194	82,802	82,894	93	31.87	8	8	<0.00001	*RMA_00027785*	Unknown
ctg6434^b^	97,065	97,333	269	21.67	10	3	0.00033	*SCNN1G*	Amiloride‐sensitive sodium channel subunit gamma
ctg6628	18,146	18,198	53	25.17	8	3	0.00016	*RMA_00047359*	Unknown
ctg6924	35,252	35,360	109	−20.87	6	3	0.0016	Intergenic	N/A
ctg7054^a^	25,391	25,575	185	29.34	11	6	<0.00001	Intergenic	N/A
ctg7585^c^	41,611	41,764	154	32.81	11	10	<0.00001	*FAM168A*	Protein FAM168A
ctg76	43,940	44,093	154	67.00	11	11	<0.00001	Intergenic	N/A
ctg7863^c^	136,569	136,654	86	−40.76	6	5	<0.00001	Intergenic	N/A
ctg7875	169,559	169,767	209	29.38	15	5	<0.00001	Intergenic	N/A
ctg7948^a^	42,453	42,704	252	39.57	11	8	<0.00001	Intergenic	N/A
ctg8148	134,671	135,196	526	27.27	32	14	<0.00001	Intergenic	N/A
ctg8148	161,076	161,507	432	−21.49	11	3	0.00005	Intergenic	N/A
ctg8148	161,900	162,142	243	−30.57	11	9	<0.00001	Intergenic	N/A
ctg892	3081	3230	150	‐29.49	5	3	0.00003	Intergenic	N/A
ctg913	1,182,605	1,183,295	691	21.84	16	3	0.0013	*NLK*	Serine/threonine‐protein kinase NLK
ctg994	348,235	348,448	214	43.47	8	6	<0.00001	Intergenic	N/A
ctg998^c^	35,687	35,889	203	−21.16	7	3	0.00001	*HYDIN*	Hydrocephalus‐inducing protein homolog

^a^ Also found between range‐core tadpoles exposed to alarm cues and controls.

^b^ Also found between range‐edge tadpoles exposed to alarm cues and controls.

^c^ Also found between range‐core tadpoles exposed to cannibal cues and controls.

**TABLE A4:** DMRs between range‐core tadpoles exposed to alarm cues and controls.

Contig	Start DMR	End DMR	Length DMR (bp)	Methylation difference (%)	No. CpGs	No. DMCs	DMR	Gene	Protein
							*q*‐Value		
ctg10314	19,548	19,677	130	−21.49	6	3	0.0015	Intergenic	N/A
ctg11043	174,618	174,666	49	33.37	7	3	<0.00001	Intergenic	N/A
ctg11822	464,547	464,739	193	28.17	11	5	<0.00001	Intergenic	N/A
ctg1295	229,245	229,311	67	23.27	5	4	<0.00001	Promoter of *HGSNAT*	Heparan‐alpha‐glucosaminide N‐acetyltransferase
ctg14203	43,476	43,558	83	39.58	6	5	<0.00001	*NXPE4*	NXPE family member 4
ctg15206	55,131	55,224	94	22.66	8	3	0.00007	Intergenic	N/A
ctg1550	233,177	233,268	92	24.80	8	4	0.00005	Intergenic	N/A
ctg1915	401,724	401,823	100	−22.27	7	3	<0.00001	Promoter of *PITPNC1*	Cytoplasmic phosphatidylinositol transfer protein 1
ctg2335	167,218	167,376	159	32.83	5	5	<0.00001	*RMA_00013998*	Unknown
ctg2404	71,060	71,357	298	−24.84	5	3	0.0004	*CEP135*	Centrosomal protein of 135 kDa
ctg3020	75,379	75,555	177	−27.84	8	4	<0.00001	*RNASEH1*	Ribonuclease H1
ctg3195^b^	95,366	95,827	462	22.17	16	12	<0.00001	*AKTIP‐A*	AKT‐interacting protein homolog A
ctg3326	438,556	438,997	442	24.00	6	5	<0.00001	Intergenic	N/A
ctg3633	303,843	304,055	213	27.85	9	4	0.00012	Intergenic	N/A
ctg3763	35,833	36,011	179	22.07	11	3	<0.00001	Intergenic	N/A
ctg399	1,149,155	1,149,333	179	26.29	11	3	0.0020	Intergenic	N/A
ctg42^c^	1,763,592	1,763,650	59	−23.52	6	5	<0.00001	*PKP4*	Plakophilin‐4
ctg4301	45,500	45,663	164	32.46	7	5	<0.00001	*ACYP2*	Acylphosphatase‐2
ctg4767	166,936	167,026	91	58.26	7	7	<0.00001	*COX10*	Protoheme IX farnesyltransferase, mitochondrial
ctg547	256,202	256,535	334	30.07	7	6	<0.00001	Intergenic	N/A
ctg552	340,264	340,358	95	24.48	7	3	<0.00001	*GRAP*	GRB2‐related adapter protein
ctg56	217,365	217,478	114	24.74	6	3	0.00007	*ARIH2*	E3 ubiquitin‐protein ligase ARIH2
ctg5947	30,168	30,324	157	21.85	10	4	<0.00001	Intergenic	N/A
ctg6147	93,558	94,000	443	−21.03	13	10	<0.00001	*CFAP77*	Cilia‐ and flagella‐associated protein 77
ctg7054^c^	25,391	25,575	185	27.43	11	5	0.00073	Intergenic	N/A
ctg7948^c^	42,373	42,704	332	25.42	13	8	<0.00001	Intergenic	N/A
ctg8084^a^	148,657	148,744	88	26.62	5	3	<0.00001	*GUCA1A*	Guanylyl cyclase‐activating protein 1
ctg8225	102,294	102,357	64	34.95	8	4	<0.00001	*ENG*	Endoglin
ctg834	307,186	307,251	66	24.57	5	4	<0.00001	Promoter of *PKD1*	Polycystin‐1
ctg9382	50,247	50,337	91	23.90	8	4	<0.00001	Intergenic	N/A
ctg9969^b^	28,668	28,807	140	27.29	5	3	<0.00001	Intergenic	N/A
ctg998	271,935	272,149	215	−34.02	6	5	<0.00001	*RMA_00013094*	Fatty acyl‐CoA hydrolase precursor, medium chain

^a^ Also found between range‐edge tadpoles exposed to alarm cues and controls.

^b^ Also found between range‐core tadpoles exposed to cannibal cues and controls.

^c^ Also found between range‐edge tadpoles exposed to cannibal cues and controls.

**TABLE A5:** DMRs between range‐edge tadpoles exposed to alarm cues and controls.

Contig	Start DMR	End DMR	Length DMR (bp)	Methylation difference (%)	No. CpGs	No. DMCs	DMR	Gene	Protein
							*q*‐Value		
ctg1044	3039	3347	309	23.47	11	10	<0.00001	*CD63*	CD63 antigen
ctg111	23,206	23,369	164	−28.05	5	4	<0.00001	Intergenic	N/A
ctg1181	1649	1851	203	21.44	12	6	<0.00001	*FCF1*	rRNA‐processing protein FCF1 homolog
ctg1181^c^	2961	3382	422	22.90	16	10	<0.00001	Intergenic	N/A
ctg1325	156,402	156,483	82	27.99	6	4	<0.00001	*PRKCZ*	Protein kinase C zeta type
ctg1428	238,871	238,986	116	21.59	6	3	<0.00001	*TIE1*	Tyrosine‐protein kinase receptor Tie‐1
ctg1543	245,121	245,213	93	38.13	5	3	<0.00001	*L1RE1*	LINE‐1 retrotransposable element ORF1 protein
ctg1563	762,370	762,895	526	−20.97	11	6	<0.00001	Intergenic	N/A
ctg1699	27,371	27,721	351	−24.68	9	4	<0.00001	Intergenic	N/A
ctg17660	14,354	14,430	77	21.85	7	6	<0.00001	Intergenic	N/A
ctg17928	59,148	59,241	94	29.58	9	8	<0.00001	Intergenic	N/A
ctg18198	77,767	78,023	257	26.07	17	6	<0.00001	*PHYH*	Phytanoyl‐CoA dioxygenase, peroxisomal
ctg1899	133,489	133,541	53	20.57	5	4	<0.00001	*MST1R*	Macrophage‐stimulating protein receptor
ctg19321^c^	2398	2512	115	21.44	7	5	<0.00001	*RMA_00054127*	Unknown
ctg21885	14,026	14,189	164	46.15	9	9	<0.00001	*ZCCHC3*	Zinc finger CCHC domain‐containing protein 3
ctg21885	15,781	15,992	212	45.18	5	5	<0.00001	*ZCCHC3*	Zinc finger CCHC domain‐containing protein 3
ctg21885	16,381	16,960	580	42.73	25	25	<0.00001	*ZCCHC3*	Zinc finger CCHC domain‐containing protein 3
ctg21885	17,460	17,545	86	32.86	7	5	<0.00001	*ZCCHC3*	Zinc finger CCHC domain‐containing protein 3
ctg21994	18,763	18,920	158	−36.07	11	9	<0.00001	Intergenic	N/A
ctg22378	15,218	15,321	104	26.00	8	5	<0.00001	Intergenic	N/A
ctg24612	52,646	52,945	300	21.05	20	11	<0.00001	Intergenic	N/A
ctg2502	121,898	121,990	93	36.61	5	5	<0.00001	Intergenic	N/A
ctg2506	228,750	228,816	67	30.41	6	3	<0.00001	Promoter of *ECE1*	Endothelin‐converting enzyme 1
ctg25970^c^	30,960	31,112	153	37.25	13	13	<0.00001	Intergenic	N/A
ctg26366	16,221	16,386	166	30.65	6	4	<0.00001	Intergenic	N/A
ctg2771	629,305	629,399	95	−28.13	8	4	0.00043	*HPCAL1*	Hippocalcin‐like protein 1
ctg2853	338,699	338,786	88	−26.29	5	4	<0.00001	*RMA_00013077*	Unknown
ctg2982	394,793	394,914	122	−23.83	10	8	<0.00001	*RIMS2*	Regulating synaptic membrane exocytosis protein 2
ctg2989	8014	8094	81	−43.56	5	5	<0.00001	*KCNK10*	Potassium channel subfamily K member 10
ctg30982	9910	9982	73	23.30	8	6	<0.00001	Intergenic	N/A
ctg3616	109,281	109,463	183	21.72	8	3	0.00010	Intergenic	N/A
ctg3705	442,058	442,262	205	35.97	7	5	<0.00001	Intergenic	N/A
ctg3707	72,464	72,610	147	21.45	9	5	<0.00001	Intergenic	N/A
ctg3758^c^	44,460	44,677	218	23.35	13	7	<0.00001	Intergenic	N/A
ctg3773^c^	120,179	120,346	168	−21.59	11	6	<0.00001	Intergenic	N/A
ctg3773^c^	120,787	121,180	394	−34.95	25	21	<0.00001	*SOCS3*	Suppressor of cytokine signaling 3
ctg3773^c^	171,024	171,542	519	−21.52	15	6	<0.00001	*PGS1*	CDP‐diacylglycerol‐glycerol‐3‐phosphate 3‐phosphatidyltransferase, mitochondrial
ctg399^b^	944,763	945,066	304	29.24	19	12	<0.00001	*RMA_00000723*	Unknown
ctg4038	34,701	34,793	93	20.37	7	4	<0.00001	Intergenic	N/A
ctg4124	248,589	248,766	178	35.49	8	5	<0.00001	*PARD6B*	Partitioning defective 6 homolog beta
ctg5424^c^	170,718	170,848	131	−27.62	5	4	<0.00001	*HSPB6*	Heat shock protein beta‐6
ctg5497	14,077	14,209	133	24.35	5	3	0.00005	*C11ORF96*	Uncharacterized protein C11orf96
ctg5507	2222	2483	262	−21.88	11	8	<0.00001	Intergenic	N/A
ctg6147	375,208	375,342	135	−22.06	12	5	<0.00001	Intergenic	N/A
ctg6344	100,935	101,066	132	−27.92	14	5	0.00004	*EPHB5*	Ephrin type‐B receptor 5
ctg6434^c^	97,065	97,333	269	20.82	10	8	<0.00001	*SCNN1G*	Amiloride‐sensitive sodium channel subunit gamma
ctg6434	98,765	98,965	201	−25.83	8	3	<0.00001	*SCNN1G*	Amiloride‐sensitive sodium channel subunit gamma
ctg6643	54,137	54,327	191	28.76	14	10	<0.00001	Intergenic	N/A
ctg6922	112,733	112,867	135	52.82	5	4	<0.00001	Intergenic	N/A
ctg8084^a^	148,657	148,744	88	28.43	5	3	<0.00001	*GUCA1A*	Guanylyl cyclase‐activating protein 1
ctg8281	36,970	37,033	64	−25.67	7	7	<0.00001	Intergenic	N/A
ctg9467	108,460	108,672	213	−24.32	5	3	0.0081	*MINK1*	Misshapen‐like kinase 1
ctg966	144,472	144,685	214	22.53	12	11	<0.00001	Intergenic	N/A

^a^ Also found between range‐core tadpoles exposed to alarm cues and controls.

^b^ Also found between range‐core tadpoles exposed to cannibal cues and controls.

^c^ Also found between range‐edge tadpoles exposed to cannibal cues and controls.
